# Improvement of right heart structure and function by BAY 41-8543 in pulmonary artery banded mice

**DOI:** 10.1186/1471-2210-11-S1-P79

**Published:** 2011-08-01

**Authors:** Wiebke Janssen, Yves Schymura, Astrid Wietelmann, Johannes-Peter Stasch, Himal Luitel, Norbert Weissmann, Hossein Ardeschir Ghofrani, Friedrich Grimminger, Thomas Braun, Werner Seeger, Ralph Theo Schermuly

**Affiliations:** 1 Max-Planck-Institute for Heart and Lung Research, Bad Nauheim, Germany; 2 University of Giessen Lung Centre (UGLC), Giessen, Germany; 3 Bayer Pharma AG, Cardiology Research, Wuppertal, Germany

## Background

Receptors for the natriuretic peptides (NPs) ANP, BNP and CNP are highly expressed in lung, suggesting that this organ is an important physiological target for NP signalling. By stimulating vaso- and bronchodilation and inhibiting cell proliferation and fibrotic processes, NPs may have therapeutic potential in various lung diseases. Mechanisms responsible for the relatively short half-life (few min) of NPs and the control of their local concentrations include (i) receptor-mediated internalization by the so called clearance receptor (NPR-C) and (ii) degradation by a membrane metalloprotease, called neutral endopeptidase (NEP) or neprilysin [1]. The velocity of peptide degradation/inactivation differs between ANP, BNP and CNP. Interestingly, cleavage of NPs by insulin-degrading enzyme (IDE) [2] may have a particular role in NP signalling by generating peptide fragments that are hyperactive in receptor stimulation [3].

The physiological significance of NEP was supported by several studies in rodents showing that NEP inhibition leads to increased NP concentrations and activity. Moreover, we found that NEP inhibition is necessary and sufficient for detection of GC-A and GC-B by affinity labelling experiments with radioactive ANP or CNP in mouse and rat lung membrane preparations. Analogous assays, however, failed to label these receptors in human lung membranes, suggesting potent NP-degrading activity of NEP inhibitor-insensitive proteases.

## Methods and results

ANP degradation by lung membranes in either the absence or presence of NEP inhibitors was analyzed by thin-layer-chromatography and mass spectrometry [2]. We found that NEP inhibition strongly reduces ANP degradation by rat and mouse but not human membranes. ANP-degrading activity in human lung membranes under conditions of NEP inhibition was very potent and even detectable with 1 ng of membrane protein. Like ANP, CNP was rapidly hydrolyzed. In both peptides, initial cleavage occurred at the same position within the conserved peptide ring structure being essential for biological activity. A second cleavage each was localized to the amino-terminus (behind Arg-4 or Lys-4, respectively). The cleavage sites are unrelated to those by NEP and IDE and indicate trypsin-like enzyme activity. Unlike ANP and CNP, BNP is a poor substrate and shows a completely different and complex cleavage pattern after prolonged incubation. The NEP inhibitor-insensitive protease was also detectable, albeit at much lower levels, in membranes from human aorta and mesenteric arteries, but not at all in placenta. Studies with various protease inhibitors revealed that leupeptin exposure potently inhibits NP degradation by this activity.

## Conclusion

A novel trypsin-like enzyme activity, but not NEP, is the major natriuretic peptide-degrading membrane protease in human lung. The accumulated expression of this enzyme in human pulmonary tissue favours potential therapeutic interventions in lung diseases. Leupeptin may act as a beneficial agent in this regard.

## References

[B1] Potter LR Abbey-Hosch S Dickey DM Natriuretic peptides, their receptors, and cyclic guanosine monophosphate-dependent signaling functions. Endocrine Rev 2006 27 47 72 16291870 10.1210/er.2005-0014

[B2] Müller D Schulze C Baumeister H Buck F Richter D The rat insulin-degrading enzyme: cleavage pattern of the natriuretic peptide hormones ANP, BNP and CNP revealed by HPLC and mass spectrometry Biochemistry 1992 31 11138 11143 10.1021/bi00160a026 1445854

[B3] Ralat LA Guo Q Ren M Funke T Dickey DM Potter LR Tang WJ Insulin-degrading enzyme modulates the natriuretic peptide-mediated signaling response J Biol Chem 2011 286 4670 4679 10.1074/jbc.M110.173252 21098034 PMC3039328

