# Correction: Improvement of right heart structure and function by BAY41-8543 in pulmonary artery banded mice

**DOI:** 10.1186/1471-2210-11-S1-P80

**Published:** 2011-09-14

**Authors:** Wiebke Janssen, Yves Schymura, Astrid Wietelmann, Johannes-Peter Stasch, Himal Luitel, Norbert Weissmann, Hossein Ardeschir Ghofrani, Friedrich Grimminger, Thomas Braun, Werner Seeger, Ralph Theo Schermuly

**Affiliations:** 1 Max-Planck-Institute for Heart and Lung Research, Bad Nauheim, Germany; 2 University of Giessen Lung Centre (UGLC), Giessen, Germany; 3 Bayer Schering Pharma, Cardiology Research, Wuppertal, Germany

## 

Abstract P79 [1] was published in error and the correct text follows:

## Background

Right heart failure is a prevalent mechanism of cardiovascular collapse and distinctly different from left heart failure. Afterload reduction has been the main focus to treat right ventricular (RV) dysfunction, but it cannot be achieved in many cases. A new strategy is to directly target increased RV hypertrophy. Pulmonary artery banding (PAB) is used to induce the RV hypertrophy, without any changes in the pulmonary vasculature. The nitric oxide (NO) pathway was shown to be crucially involved in the development of left ventricular hypertrophy. In this study we assessed the effects of the sGC stimulator BAY 41-8543, the PDE5 inhibitor sildenafil, and combination treatment on RV function and RV hypertrophy.

## Methods

Chronic pressure-overload RV hypertrophy was induced by PAB in male C57/Bl6 wild-type mice. Drug treatment was started seven days after surgery for the duration of 2 weeks, after which the consequences of the sustained pressure overload on RV morphology and function were assessed using Magnetic Resonance Imaging (MRI) and RV catheterization. Furthermore, fibrosis was assessed by histology.

## Results

Three weeks after PAB, placebo-treated mice showed several signs of RV dysfunction as compared to sham-operated mice. PAB led to RV systolic dysfunction, as indicated by an increase in end-systolic volume (17.3±1.0 ml vs. 29.2±2.5 ml [Sham vs. PAB]), decreased RV stroke volume (40.5±2.3 ml vs. 23.0±3.3 ml) and decreased RV ejection fraction (70.0±1.0 % vs. 43.0±3.5 %). Treatment with sildenafil did neither lead to significant changes in RV end-systolic volume, RV stroke volume nor RV ejection fraction, whilst treatment with BAY 41-8543 and combination treatment led to significant improvements. (RV end-systolic volume: 29.2±2.5 vs. 23.6±3.3 vs. 24.6±1.1 vs. 19.1±3.8; RV stroke volume: 23.0±3.3 vs. 27.3±2.3 vs. 32.1±3.8 vs. 31.8±1.3; RV ejection fraction: 43.3±3.5 vs. 54.1±3.0 vs. 55.7±3.7 vs. 63.8±4.4 [all values as %; placebo vs. sildenafil vs. BAY 41-8543 vs. combination treatment]). PAB mice showed RV hypertrophy (0.022±0.003g vs. 0.032±0.006g) and an increased RV/(LV-S) ratio (0.25±0.03 vs. 0.39±0.1). Drug treatment had no effects on either RV hypertrophy nor RV/(LV+S) ratio. RV pressure was increased in PAB mice (30.0±3.0 mmHg vs. 50.8±12.2 mmHg) and did not change under drug treatment. Histological assessment of fibrosis showed that the collagen content increased in banded mice, sildenafil had no effects on collagen content, and BAY 41-8543 and combination treatment both decreased the amount of collagen (7.6±2.1 vs. 1.2±0.1 8.8±5.8 vs. 3.3±1.2 vs. 3.3±1.2 [all values as %; placebo vs. sham vs. sildenafil vs. BAY 41-8543 vs. combination treatment]).

## Conclusions

Even though none of the drug treatments led to significant changes in RV mass, BAY 41-8543 and combination treatment with sildenafil led to significant improvements in RV function, accompanied by decreased fibrosis.
